# Photon- and Proton-Mediated Biological Effects: What Has Been Learned?

**DOI:** 10.3390/life13010030

**Published:** 2022-12-22

**Authors:** Enar Jumaniyazova, Daniil Smyk, Polina Vishnyakova, Timur Fatkhudinov, Konstantin Gordon

**Affiliations:** 1 Research Institute of Molecular and Cellular Medicine, Peoples’ Friendship University of Russia (RUDN University), Miklukho-Maklaya Street 6, 117198 Moscow, Russia; 2 A. Tsyb Medical Radiological Research Center, Branch of the National Medical Research Radiological Center of the Ministry of Health of the Russian Federation (A. Tsyb MRRC), 4, Korolev Street, 249036 Obninsk, Russia; 3 Laboratory of Regenerative Medicine, National Medical Research Center for Obstetrics, Gynecology and Perinatology Named after Academician V. I. Kulakov of Ministry of Healthcare of Russian Federation, 117997 Moscow, Russia; 4 Laboratory of Growth and Development, Avtsyn Research Institute of Human Morphology of FSBI “Petrovsky National Research Centre of Surgery”, 117418 Moscow, Russia

**Keywords:** radiation therapy, photon irradiation, proton irradiation, head and neck tumors, immune cells, tumor microenvironment

## Abstract

The current understanding of the effects of radiation is gradually becoming broader. However, it still remains unclear why some patients respond to radiation with a pronounced positive response, while in some cases the disease progresses. This is the motivation for studying the effects of radiation therapy not only on tumor cells, but also on the tumor microenvironment, as well as studying the systemic effects of radiation. In this framework, we review the biological effects of two types of radiotherapy: photon and proton irradiations. Photon therapy is a commonly used type of radiation therapy due to its wide availability and long-term history, with understandable and predictable outcomes. Proton therapy is an emerging technology, already regarded as the method of choice for many cancers in adults and children, both dosimetrically and biologically. This review, written after the analysis of more than 100 relevant literary sources, describes the local effects of photon and proton therapy and shows the mechanisms of tumor cell damage, interaction with tumor microenvironment cells and effects on angiogenesis. After systematic analysis of the literature, we can conclude that proton therapy has potentially favorable toxicological profiles compared to photon irradiation, explained mainly by physical but also biological properties of protons. Despite the fact that radiobiological effects of protons and photons are generally similar, protons inflict reduced damage to healthy tissues surrounding the tumor and hence promote fewer adverse events, not only local, but also systemic.

## 1. Introduction

With the discovery of X-rays by Wilhelm Roentgen in 1895, radiation therapy (RT) almost immediately became one of the main tools of treatment for a wide range of malignant neoplasms [1]. The effects of irradiation are based on the induction of damage to genetic apparatus, which disrupts the normal course of cell division cycle and ultimately promotes cell death. Historically, scientific interest has been predominantly focused on studying the influence of irradiation on tumor cells, while the collateral effects on peritumoral regions were largely ignored.

Current understanding of the effects of irradiation is gradually expanding. However, it is still unclear why RT provides beneficial response in some patients, but fails to halt disease progression in others. Apart from the effects of RT in tumor per se, consideration should be given to its effects in peritumoral regions and ultimately on systemic level [2].

Another important aspect to consider with regard to RT effectiveness is its influence on the immune system [3]. Recent findings indicate that, apart from its local effects, irradiation is capable of inducing systemic responses through promotion of the synthesis of tumor-associated antigens, pro-inflammatory cytokines and chemokines [3,4]. On a par with such an inducing role in immune response, various types of ionizing radiation can also contribute to immunosuppressive cytokine production, especially upon repeated exposure. In addition, irradiation of bone marrow, blood and drainage lymph nodes has been shown to promote severe leukopenia and lymphocyte damage strongly associated with poor quality of life and reduced survival [3,4]. Certain tumors enclose themselves in immune-suppressive environments favoring tumor growth and disease progression [4]. Increasing number of studies consider not only the tumor microenvironment but also the peritumoral zones for their roles in determining such phenomena as radio-resistance and relapses and as potential targets for therapeutic interventions [5,6].

Early experimental studies in this field were mostly devoted to unraveling the mechanisms of destruction of tumor cells by ionizing radiation, and the research interests were focused on radiation-induced biological effects in tumor cells [2,5]. Despite the substantial technical progress in the field of RT, its main goal of achieving the maximum lethal effect on tumor cells at minimal damage to healthy tissues can hardly be considered accomplished [3,7,8].

Proton therapy (PT) is one of the most promising types of RT and its widespread use is expected to address many of the above-mentioned problems. The accumulating clinical experience, ahead of fundamental radiobiological research in many aspects, has already promoted a steady increase in the number of proton centers around the world [9]. Although rigorous studies on local and systemic effects of PT are still rare, the available evidence links the favorable profiles of proton irradiation to the recognized dosi-metric advantages of charged particles and expands the understanding of their biological effects.

**Figure 1:**
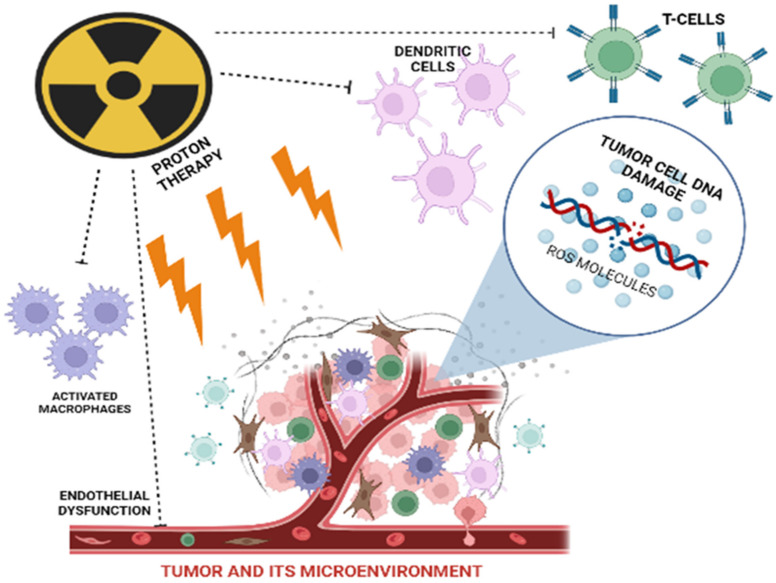
Targets for proton therapy.

This review, written after the analysis of more than 100 relevant literary sources, describes the effects of classical photon beam treatment on tumor surroundings, while discussing systemic effects of irradiation and summarizing available data on the corresponding effects of protons (Figure 1).

## 2. Local and Systemic Effects of Photon Irradiation

Photon beams constitute the main source of ionizing irradiation applied in RT. Such beams represent a type of electromagnetic radiation that, when passing through living tissues, knocks electrons out of molecules inside the cells. Direct and indirect effects of ionizing radiation are distinguished as follows: direct effects involve ionization or excitation of macromolecules by photons, whereas indirect damaging effects to cellular structures are mediated by the products of water radiolysis [10].

Ionizing radiation beams introduce single- or double-strand breaks into DNA molecules [11]. The overall number and density of double-strand breaks depend on the linear energy transfer (LET) of particular type of radiation [8,12]. Specifically, low-LET beams (photon beams such as gamma and X-rays) produce scattered DNA damage, whereas protons (in some ways high-LET) and carbon ions (high-LET beams) produce clustered i.e., heterogeneously distributed and densely positioned breaks [11].

The role of the innate immune system in malignant neoplasm progression has become a major research focus with the discovery of immunomodulatory myeloid cells inside and around the tumors. These myeloid cells, known to play a central role in the suppression of adaptive immunity, comprise several subpopulations specifically involved in tumor development. Tumor microenvironment (TME) is a complex milieu composed of blood and lymph vessels, immune cells, fibroblasts, signaling molecules (cytokines, growth factors, hormones, etc.) and extracellular matrix. Specific cellular environments comprising lymphocytes and macrophages with immunosuppressive phenotypes have been characterized as a factor of tumor progression. Besides their role in tumor progression and metastasis, such microenvironments influence tumor responses to various therapies [13,14].

Indeed, radiation exerts pleiotropic effects on tumors and their stroma, still understudied. It should be stressed that early and late responses of normal tissues to radiation are dose-limiting factors of radiation therapy that affect the therapeutic efficacy and quality of life in cancer patients. Given the involvement of tumor microenvironments in the processes of recurrence and radio-resistance development, it is often considered as a potential target for preventing these adverse phenomena [5].

Dendritic cells (DCs) show stable resistance to radiation-induced apoptosis with moderate changes to cell surface phenotype and retention of the overall capacity of migration and endo-/phagocytosis. In a study by Merrick et al. (2005), irradiated DCs were less efficient in mixed lymphocyte reaction assays and produced less IL-12 upon maturation compared to non-irradiated controls [14], while preserving secretion of IL-10 [15,16]. In vivo, adoptive transfer of DCs in the aftermath of chemo/proton combination therapy facilitated full resorption of experimentally induced tumors [3].

Macrophages are the main cellular component of tumor microenvironment, independently of the tumor localization [14,16,17,18,19]. Macrophages can either stimulate or suppress carcinogenesis, tumor invasion and metastasis depending on their functional status. Tumor cells exhibit a capacity to recruit monocytes from peripheral blood and ensure their activation into tumor-supporting phenotypes [20,21]. High densities of such pro-carcinogenic anti-inflammatory polarized macrophages in tumor microenvironments have been associated with tumor progression [22]. Accordingly, tumor-associated macrophages represent a prospective model for anti-tumor therapy. In the meantime, macrophages are one of the most radiation resistant human cell types [23], which is due to their capacity of massive production of anti-oxidant molecules including manganese superoxide dismutase (MnSOD), a scavenger of superoxide ions. The high expression levels of MnSOD endow macrophages with high tolerance to damaging effects [22,23]. Depending on specific microenvironmental features of the particular tumor (available niches and the spectrum of stimulatory factors), irradiation can enhance either anti-tumor or pro-tumor properties (or both) of tumor-associated macrophages. Seminal research by Lambert et al. (1987), with irradiated human macrophage cell lines showing enhanced cytolytic activity, has been fully supported by a host of recent finding [23,24,25]. For instance, Shan et al. (2007) and Shiga et al. (2015) demonstrate that whole-body low-dose irradiation (<1.5 Gy) enhances TLR4/MD2 and CD14 expression in murine peritoneal macrophages, thereby promoting secretion of anti-tumor cytokines (IL-12, IL-18), which indicates an increase in anti-tumor potential of these cells. Incidentally, these effects were considered a basis for a certain optimism regarding the low-dose lung irradiation during coronavirus pandemic [23,25,26].

Despite irradiation capacity to stimulate cytolytic activity and anti-tumor cytokine production in macrophages, a parallel line of evidence supports the ability of macrophages to stimulate the development of radio-resistance in a tumor. For instance, CD11b+ myeloid cells, a proportion of which are macrophages, facilitate production of growth factors, such as vascular endothelial growth factor (VEGF) and matrix metalloproteinase-9 (MMP-9), which support angiogenic programs in growing tumors. The halt of CD11b+ myeloid cell influx after RT augments the curative effect, apparently due to the concomitant drop in concentrations of iNOS and arginase I, which inhibit T cell responses [16,27,28]. Thus, despite the stimulation of macrophage cytolytic activity and anti-tumor cytokine production by ionizing irradiation, the treatment may prove insufficient for the tumor growth inhibition, if accompanied by macrophage recruiting to the tumor and/or predominance of macrophage polarization towards pro-tumor phenotypes [15,16]. 

Natural killer (NK) cells play an important role in anti-tumor immunity by attacking malignant cells through direct cytolysis and massive secretion of immune mediators including several cytokines and chemokines [29]. Tumor cell exposure to ionizing radiation in vitro promoted them from expressing specific ligands to NKG2D—an activating receptor on NK cells [30,31].

Other proinflammatory stress molecules released by dying cells include heat shock protein 70 (Hsp70), a stress response protein responsible for binding defective proteins and presenting them on the cell surface [32,33]. When irradiated, pancreatic and colon carcinoma cells release the Hsp70 protein, which is recognized by NK cells, making them lyse the cancer cells [16]. The NKG2D ligands and Hsp70 make the cells more vulnerable to NK cell cytolysis, which indicates that the RT-stimulated NK activity may be an important predictor of response to the therapy.

To preserve their vital capacity, tumor cells maintain dynamic interactions with their microenvironment. To facilitate formation of the stroma, tumor cells secrete regulatory molecules that support proliferation and recruitment of stromal cells from nearby regions into the tumor.

Fibroblasts, which constitute the basis of the stroma in both normal and transformed tissues, are heterogeneous by origin, as they differentiate from a variety of precursors (mesenchymal stem cells, fibrocytes, pericytes and epithelial cells formed by epithelial-mesenchymal transition). In tumors, fibroblasts comprise diverse subpopulations collectively termed ‘tumor-associated fibroblasts’ (TAFs). The distinguishing feature of these cells is their constant activity and the lack of apoptosis as in normal fibroblasts [25,34]. They produce extracellular matrix proteins (e.g., tenascin C and collagen type I), cytokines (e.g., hepatocyte growth factor, HGF; platelet-derived growth factor, PDGF; CXC chemokine ligand 12, CXCL12, a.k.a. stromal cell-derived factor 1) and matrix-remodeling enzymes (notably metallopeptidases).

In terms of functional characteristics, TAFs act as synergists of tumor cells, creating an immunosuppressive network that helps to rid the tumor of the immune-mediated destruction. Overall, TAFs are thought to play a key role in tumor progression and their high content in the tumor microenvironment has been associated with poor prognosis [34].This is primarily due to their ability to endure various stressors, such as chemotherapy and radiotherapy. Thus, TAFs are a resistant cell type that can actively promote tumor recurrence [35,36,37]. 

Chronic inflammation is the principal initiating factor of fibrosis, with the persistent immune responses accompanied by tissue remodeling and repair processes. Prevention of fibroproliferative processes, important for the normal organ function maintenance, is crucial for improved quality of life after radiotherapy [5]. The rare dedicated studies considering RT effects on stromal cells almost invariably involve TAFs freshly isolated from human tumor tissues. As demonstrated by Hellevik et al. (2012), TAFs isolated from non-small cell lung cancer samples survived both single-dose irradiation (2, 6, 12 or 18 Gy) and fractionated regimens (6 fractions of 3 Gy) while losing their invasive potential [36]. None of the tested irradiation regimens interfered with cell survival in the course of 3 weeks after treatment. According to anti-53BP1 staining data, the irradiation caused DNA damage in TAFs in a dose-dependent manner (18 Gy > 12 Gy > 6 Gy >2 Gy). Moreover, a single 18 Gy dose (ablation dose) caused persistent DNA damage, by contrast with the 6 × 3 Gy regimen. In addition, β-galactosidase staining revealed a more pronounced senescence reaction in TAFs in response to single-dose irradiation compared to fractionated regimen.

While a number of studies claim that RT negatively affects fibroblasts through growth arrest and cell senescence, others suggest that it promotes activation of normal fibroblasts by inducing a senescent-like phenotype [37]. Fibroblasts with this phenotype differ from the conventional replicative-senescent cells in lacking the telomere shortening. Thus, they have characteristics similar to activated fibroblasts and may be regarded as TAF-like cells [38,39].

Recent studies demonstrate that secreted factors produced by TAFs induce chemotherapy resistance in tumor cells [39,40,41,42]. The multiple direct and indirect roles of TAFs in chemotherapy resistance alongside research evidence strongly suggest their participation in RT [42].

Studies focused on the direct cytotoxic effects of fractionated RT on TAFs have shown that the fibroblasts are naturally resistant to radiation [38,39]. Tommelein et al. (2018) irradiated TAFs from colorectal cancer (5–10 fractions, 1.8 Gy each). The irradiation caused DNA damage, p53 activation and cell cycle arrest in TAFs. However, none of the regimens caused overt cell death or morphological changes [43,44].

These results indicate that RT does affect TAF proliferation at the molecular genetic level, but leaves the cells viable to maintain a microenvironment that can promote the growth of resistant tumor cells [38,39].

Another source of influence on tumor microenvironments is the capacity of photon beams to potentiate angiogenesis and lympho-genesis by stimulating the release of growth factors and other signaling molecules, notably VEGF, IL-6 and IL-8.

A study on a canine oral melanoma cell line demonstrated the irradiation-mediated potentiation of VEGF release by tumor microenvironment cells. Moreover, the effect was quantitative, with an increase in a single focal dose and irradiation time lapse positively affecting VEGF secretion levels [45].

Pasi et al. (2010) studied the influence of irradiation on IL-6 and IL-8 production levels in human glioblastoma cells. The application of ionizing radiation to human glioblastoma cell line promoted an increase in IL-6 and IL-8 secretion by these cells. The authors reckon that hyperproduction of interleukins can represent a protective reaction of the tumor to the therapy and a signal to initiate tumor spread to surrounding healthy tissues [46].The concomitant stimulation of angiogenesis and lympho-genesis in irradiated healthy tissues actualizes seeking the means for more focused irradiation of the tumor, for example, through the use of PT.

RT interactions with the components of tumor microenvironment, especially vasculature, are being extensively studied [47,48]. Endothelial cells of tumor microenvironment are highly proliferative, which augments their sensitivity to irradiation. Each endothelial cell supports the growth of about 2000 cancer cells, and the reaction of endothelium to irradiation, including survival and recovery mechanisms, is a priority research focus in cancer studies [5,48].

**Figure 2:**
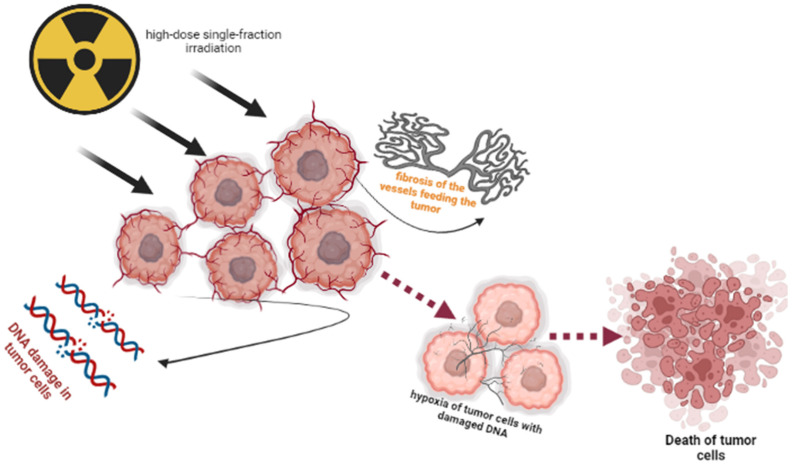
Tumor cell death induced by high-dose single-fraction irradiation.

Understanding the effect of radiation on the functional state of tumor microvasculature is important for improving the effectiveness of RT. The RT-induced alterations of the tumor vascular bed depend on the total dose and fractionation regimen, as well as type, location and stage of the tumor and specific features of vascular morphology (wall structure). RT causes endothelial cell dysfunction manifested by increased permeability, detachment from the basement membrane and apoptosis. High single doses (8–16 Gy) have been associated with increased levels of acid sphingomyelinase (ASMase), which induces endothelial cell apoptosis [5,49,50]. (Figure 2). The RT-induced endothelial cell dysfunction and apoptosis promote local inflammation and fibrosis. Within the lumina, irradiation promotes a pro-thrombotic state characterized by platelet aggregation, formation of microthrombi, and increased adhesion of pro-inflammatory cells to the endothelium, followed by diapedesis to perivascular space [51].

From a structural perspective, irradiation facilitates destruction of blood vessels in a dose-dependent manner, especially small ones [47]. The reduction in vascular density leads to insufficient perfusion of the tumor. Long-term effects of irradiation are characterized by thickening of the vascular intima, a tendency towards sclerosis and possibly other delayed morphological alterations including thrombosis, fibrosis and median necrosis [52,53,54]. Subsequent tumor revascularization occurs via hypoxia-inducible factor 1α (HIF1α)-dependent and HIF1α-independent recruitment of bone marrow-derived cells (BMDCs) [5,55].

Overall, irradiation represents a powerful inducer of vascular damage, inflammation and fibrosis. Hypoxia and the activation of HIF1α/VEGF signaling through radiation-induced vascular dysfunction may contribute to radio-resistance development [55]. The treatment may also trigger massive inflammatory and fibrotic reaction of the stroma orchestrated by cytokines including IL-1, IL-6, IL-10 and TGF-β, which can alter the tumor response to both radiation and chemotherapy [56].

Importantly, single-fraction high-dose irradiation (15–20 Gy) can irreversibly suppress the blood flow facilitating an irreversible change in vasculature architectonics of the tumor parenchyma [5,57].

RT facilitates control of the tumor process not only through direct action on the focus, but also by mobilizing various mechanisms of activation and suppression of the immune system [44]. One of the main routes involves the ability of photon beams to enhance expression of various molecules on the surface of tumor cells, including cell adhesion molecules, programmed cell death receptors, stress-induced ligands and immunostimulatory molecules [58]

In particular, photon therapy directly stimulates T-cell immune response by enhancing expression of the main histocompatibility complex class I (MHC-I) molecules by the tumor [59]. An important factor in the activation of the immune response are damage-associated molecular patterns (DAMPs) including proteins, DNA, lipids and cell fragments released to extracellular spaces upon cell death resulting from radiation exposure [60,61,62]. Information on key types and functions of DAMPs relevant to tumor growth, metastasis and response to therapy is given in Table 1.

In addition to direct mechanisms of immunity activation, there is also an indirect, so-called bystander effect. It consists in the transfer of DAMPs and other molecules that activate apoptosis or cell death from irradiated to neighboring healthy cells through connecting channels. When these molecules enter a healthy cell, apoptosis can be induced in a cell that has not been exposed to ionizing radiation [44]. However, this bystander effect can also play an opposite role, protecting non-irradiated cells and stimulating repair mechanisms in irradiated tissues due to their interactions with neighbors [81,82].

Another mechanism of immune system potentiation involves the so-called abscopal effect—the ability of local irradiation to induce an anti-tumor response in areas to which no RT has been applied. The effect is based on the release of tumor antigens upon the ionizing radiation-induced cell death. The released antigens are eventually captured by antigen-presenting cells (notably DCs) and their subsequent presentation to T cells facilitates the formation of specific anti-tumor immunity [83]. Despite the skepticism expressed by some experts regarding the clinical reality of the abscopal effect, Grimaldi et al. (2014) published a study enrolling 21 melanoma patients with metastases of varying localization after progression during therapy with ipilimumab at a dose of 3 mg/kg. The abscopal effect was recorded in 11 patients, invariably following a response to previously performed local irradiation. The median survival was 22.4 months in the ‘abscopal’ group and only 8.3 months in the no-effect group (*p* = 0.002) [84].

Apart from its immunomodulatory effect, photon therapy also exerts an immunosuppressive effect [62]. Using human DC cultures, Merrick et al. demonstrated that cells treated with ionizing radiation released much smaller amounts of IL-12 than non-treated control cultures. Besides, the irradiated DCs were much less efficient inducers of the naïve T lymphocyte differentiation than non-irradiated controls and the resulting cytotoxic lymphocytes had lower antigen-destruction capacity [15].

In addition, cells damaged by photon therapy are capable of secreting immunosuppressive cytokines and signaling molecules including IL-10, TGF-β and prostaglandin E2. IL-10, released from damaged cells, interacts with tumor microenvironment to reduce the antigen presentation rates and NK cell counts, suppressing the Th1-mediated responses thereby alleviating the monocyte and macrophage functionalities [85]. As revealed by other studies, IL-10 reduces the rates of DC maturation [62]. The use of photon therapy increases the amount of prostaglandin E2 in tumor microenvironment. Released by the dying tumor cells, prostaglandin E2 inhibits the cytotoxic lymphocyte functionalities and differentiation rates while stimulating T suppressor formation [86].

TGF-β plays an important role not only in immunosuppression, but also in oncogenesis. After the death of a tumor cell, TGF-β is released into surrounding tissues to exert a negative effect on the specific immune response by increasing the counts of T suppressors while blocking the lymphocyte and macrophage activation [87].

## 3. Local and Systemic Effects of Proton Irradiation

It has been generally accepted that the therapeutic effect of PT is comparable to photon irradiation and involves substantial damage to genetic material in tumor cells, causing disruption of cell cycle and tumor cell death [88,89]. On their way towards the target, the protons induce various types of DNA damage including nucleotide base modifications, abasic sites and single-strand breaks, the latter being the most common type of PT-induced direct damage. The irradiation also causes cytotoxicity indirectly through the reactive oxygen species (ROS) formation [89]. PT exposure facilitates massive production of ROS. At the same time, proton beams are strong inducers of apoptosis due to effective cleavage of caspase-3 specifically by protons [90].

Proton irradiation facilitates the release of DAMPs, triggering a cascade of antigen-presenting cell (APC) activation [89,91]. The immunological alarm signals, e.g., HMGB1, are transmitted by primary CD8+ T cells through activation of Toll-like receptors on APCs [68,92]. T cells can subsequently develop memory responses against the tumor. Proton irradiation can also enhance the expression of MHC class I by tumor cells for antigen presentation and the release of pro-inflammatory chemokines that attract other APCs and cytotoxic T lymphocytes (CTLs) [93,94]. The PT-induced release of tumor antigens also stimulates the migration of APCs to the draining lymph nodes in order to augment the priming of T cells for CTL-dependent systemic response initiation [95]. Cross-presentation of antigens released by DCs in the tumor microenvironment represents another effect of local PT promoting tumor eradication, which highlights the importance of cross-presentation of tumor antigens by MHC-II expressing APCs, in addition to direct presentation by MHC-I on tumor cells during CTL formation. The presence of CTL before PT initiation of correlates with better survival for different tumor types [92].

As demonstrated in vitro by Lupu-Plesu et al. (2017), proton irradiation inhibits expression of factors involved in lympho- and angiogenesis, inflammation and immune tolerance by head-and-neck squamous cell carcinoma (HNSCC), which implicates survival of less aggressive tumor cell phenotypes after proton irradiation [96]. Cells surviving three cycles of proton irradiation showed downregulated expression of pro-angiogenic/pro-inflammatory genes, except *VEGF-c*, by contrast with their activation by photons on a par with other regulatory genes. These results explain the reduced rates of lymphangio-genesis and metastasis in the aftermath of PT, as compared with conventional RT. Thus, proton and photon beams differently modulate the expression of pro-inflammatory genes in HNSCC cells. IL-8 showed the highest mRNA levels among the studied genes. Cellular stress and the therapy-induced IL-8-mediated signaling were identified as the main factors of tumor cell resistance [97]. IL-8 concentration has a significant impact on disease-free survival in patients with early-stage HNSCC [98]. In this regard, understanding the possibilities of IL-8 signaling inhibition upon proton irradiation may have considerable therapeutic value.

By contrast with photon irradiation, proton beams suppress IL-6 expression at mRNA level. IL-6 expression has been associated with poor response to chemoradiotherapy and poor prognosis in patients with HNSCC [96], as well as radiation resistance and chronic post-irradiation toxicity development [99]. These findings identify PT as a highly promising approach in HNSCC treatment, as the conventional photonic options have already reached their technical limits [38,100,101].

PT has also been shown to induce the immunogenic death of tumor cells, mainly attributed to the recruited APCs (dendritic cells) which initiate processing of tumor-associated antigens and cross-presentation of antigenic peptides at major histocompatibility complex class I (MHC I) in response to irradiation [102,103]. Cross-presentation of tumor antigens promotes the tumor-specific activation of T cells infiltrating the tumor.

Another possible irradiation-induced activation scenario involves type I interferon (IFN) production, which enhances both DC and T cell functionalities, complemented and reinforced by the direct effect of PT, which consists in DNA damage. Fragments of damaged DNA can enter the cytosolic compartment and independently enhance the IFN-mediated DC recruitment for cross-presentation of tumor-associated antigens [104,105,106].

The rigorous evidence on immunomodulatory potential of proton beams is sparse, but their immunostimulatory properties are reportedly higher [107,108] or comparable [109] to those of photons. Although the correlation between the high LET of charged particle irradiation and their immunomodulatory potential is still debated, the increased dose-dependence combined to the independence from tumor oxygenation represent undeniable advantages, especially for the treatment of hypoxic neoplasms such as breast cancer and pancreatic duct adenocarcinoma [110,111]; still, these effects are more pronounced with densely ionizing beams [112] than protons. At the same time, PT can exert an opposite, immunosuppressive influence by inducing the tumor infiltration with regulatory T cells (T-regs) or stimulating the immunosuppressive effector immunity encompassing macrophages and other immune cells of myeloid origin [105,113].

As can be seen from the above, the biological effects of PT are identical to RT (Table 2), but because of the physical and biological parameters it causes more permanent damage to the DNA of tumor cells.

## 4. Conclusions

To-date, photon irradiation has found wide application in clinical practice and our understanding of its effects on tumors and their environments is constantly updated [114]. Photon irradiation has found wide recognition in clinical practice and our understanding of its effects on tumors and their environments is constantly updated.

PT has potentially favorable toxicological profiles compared to photon irradiation, primarily due to physical properties of protons. Although biological effects of protons and photons are generally similar, protons inflict reduced damage to healthy tissues surrounding the tumor and hence promote fewer adverse events, not only local, but also systemic. A retrospective study by Uemura et al. (2022) demonstrates significant reduction in acute gastrointestinal toxicity for pediatric CNS tumors treated with proton therapy [115]. Consistently with these findings, Lautenschlaeger et al. (2019) show that proton irradiation of children with mediastinal Hodgkin’s lymphoma results in significantly lower doses for almost all organs at risk, while being associated with a reduction in long-term side effects in children and adolescents [116].

Minimization of adverse events improves the quality of life and has great impact on overall survival. Another notable distinction of proton therapy is reduced likelihood of early or late radiation complications in normal tissues, including secondary radiation-induced malignancies, which is especially important for young reproductive age patients and children [117]. Due to these advantages, both dosi-metric and biological, PT is increasingly already regarded as the method of choice for several adult and for all pediatric cancers. Considering the costs of treatment for late toxic effects, PT represents a reasonable and occasionally the most cost-effective option for solid tumors of various localization, as compared with conventional RT protocols [118].

Increased understanding of the biological effects of proton therapy will allow us to understand the mechanisms of interaction between protons and cancer cells and with cells in the tumor microenvironment, which will ultimately lead to optimized treatment strategies for cancer patients.

Basic research on the local and systemic effects of PT will allow this type of radiation therapy to be introduced into the clinical practice of oncologists and radiotherapists.

## Figures and Tables

**Table 1:** Types and functions of DAMPs.

DAMP Type	Function/Description	Effect on Tumor/Microenvironment
HMGB1 (amphoterin)	Cytokine mediator secreted by macrophages and monocytes or released during cell and tissue necrosis [63,64]	Stimulates the development of chronic inflammation by supporting neutrophil proliferation. Interacts with TLR4, TLR9 and RAGE receptors thereby promoting chemoresistance and survival of tumor cells [65,66,67] The interactions with TLR4 and RAGE stimulate DC migration and potentiate antigen presentation [68,69]
Calreticulin	Calcium-binding protein whose representation at the cell surface increases following irradiation, facilitating immune responses [64]	Calreticulin contained in extracellular spaces acts as a prophagocytic signal for DCs and promotes the IL-6- and TNF-mediated Th17 priming [70]; it also interacts with NY-ESO-1, an antigen with pronounced immunogenicity, which confirms the immunogenic function of calreticulin [71]
Adenosine triphosphate (ATP)	Nucleoside tri-phospate, the “energy currency” of the cell	Upon cell death or sublethal damage, it interacts with purinergic receptor P2 and stimulates formation of pro-IL-1β and pro-IL-18 which interact with macrophages to further activate non-specific immunity. ATP binding to purinergic receptors facilitates antigen presentation to T cells by DCs, differentiation of CD4+ T cells into Th1, Th2, Th17 and T suppressors along with cytotoxic lymphocyte maturation [72]
Interferons type I	Cytokines of innate and adaptive immunity [73]	Released by tumor cells to potentiate the activity of immune cells (macrophages, NK cells, cytotoxic T cells) while stimulating MHC-I expression [73]
S100 proteins	A group of low-weight calcium-binding proteins participating in inflammatory reactions and cellular homeostasis	Bind various RAGE receptors to stimulate inflammation, angiogenesis, tumor progression and metastasis through MAPK and NF-κB pathway activation [74,75,76,77]
Uric acid	A product of protein decay released to the microenvironment upon cell death	High concentration of uric acid in intercellular spaces is associated with enhanced migration of tumor cells [78] At the same time, release of uric acid by cells after chemotherapy or RT promotes tumor regression through stimulation of antigen presentation by DCs [79,80]
Annexin A1/FPR1	Phospholipid-binding protein expressed in many tissues and cell types including leukocytes and epithelial cells	Increased expression of Annexin A1 has been associated with increased tumor sensitivity to chemotherapy [79,80]

**Table 2:** Brief summary of main biological effects of photon and proton therapies.

Assessment Parameters		Photon Irradiation	Proton Irradiation
Physical properties		Photon beams deposit a high dose near the body surface and emit energy on their way to the “target” [10,11]	Proton beams deposit a relatively low dose near the body surface and emit maximum energy immediately before reaching the target [88,89]
DNA		Homogeneously spread DNA damage [11]	Clustered DNA damage: the irradiation confers cytotoxicity directly by introducing breaks in DNA and indirectly through ROS formation [10]
Stromal cells		Inhibiting cell growth; cell cycle arrest through DNA damage and p53 activation [44]	
Immune cells of tumor/peritumoral microenvironments	Macrophages	Stimulation of the cytolytic activity of macrophages and anti-tumor cytokine production [22,23,24]	
	NK cells	Photons induce expression of ligands for NKG2D—a major activating receptor on NK cells [29,30,31]	
	Dendritic cells	Suppressed secretion of IL-12, increased secretion of immunosuppressive cytokines (IL-10, TGF-β) and prostaglandin E2 [14,15]	Stimulation of active processing of tumor-associated antigens. Stimulates APC migration to draining lymph nodes, where T cell priming is enhanced to initiate a CTL-dependent systemic response
	T cells	Induction of T cell immune responses [5,73]	
	Endothelium	Endothelial cell dysfunction and apoptosis [5,51,63]	
Angio/lympho-genesis		Potentiation of angio- and lympho-genesis by stimulation of VEGF, IL-6 and IL-8 secretion [5,47,51]	Proton irradiation inhibits expression of factors that promote lympho-/angiogenesis, inflammation and immune tolerance, thus favoring survival of less aggressive tumor cell phenotypes after proton irradiation [96,97]

## Data Availability

Not applicable.

## References

[1] Delaney G., Jacob S., Featherstone C., Barton M. (2005). The role of radiotherapy in cancer treatment. Cancer.

[2] Zegers C.M.L., Rekers N.H., Quaden D.H.F., Lieuwes N.G., Yaromina A., Germeraad W.T.V., Wieten L., Biessen E.A.L., Boon L., Neri D. (2015). Radiotherapy Combined with the Immunocytokine L19-IL2 Provides Long-lasting Antitumor Effects. Clin. Cancer Res..

[3] Zhai D., An D., Wan C., Yang K. (2022). Radiotherapy: Brightness and darkness in the era of immunotherapy. Transl. Oncol..

[4] Golden E.B., Frances D., Pellicciotta I., Demaria S., Barcellos-Hoff M.H., Formenti S.C. (2014). Radiation fosters dose-dependent and chemotherapy-induced immunogenic cell death. Oncoimmunology.

[5] Barker H.E., Paget J.T.E., Khan A., Harrington K. (2015). The tumour microenvironment after radiotherapy: Mechanisms of resistance and recurrence. Nat. Rev. Cancer.

[6] Wang Z., Tang Y., Tan Y., Wei Q., Yu W. (2019). Cancer-associated fibroblasts in radiotherapy: Challenges and new opportunities. Cell Commun. Signal..

[7] Bhattacharya S., Asaithamby A. (2017). Repurposing DNA repair factors to eradicate tumor cells upon radiotherapy. Transl. Cancer Res..

[8] Kumari S., Mukherjee S., Sinha D., Abdisalaam S., Krishnan S., Asaithamby A. (2020). Immunomodulatory Effects of Radiotherapy. Int. J. Mol. Sci..

[9] Tian X., Liu K., Hou Y., Cheng J., Zhang J. (2017). The evolution of proton beam therapy: Current and future status (Review). Mol. Clin. Oncol..

[10] Tubiana M. (1973). Clinical data and radiobiological bases for radiotherapy. Curr. Top. Radiat. Res. Q..

[11] Ackerman L.V. (1972). The pathology of radiation effect of normal and neoplastic tissue. Am. J. Roentgenol. Radium Ther. Nucl. Med..

[12] Carter R.J., Nickson C.M., Thompson J.M., Kacperek A., Hill M.A., Parsons J.L. (2018). Complex DNA Damage Induced by High Linear Energy Transfer Alpha-Particles and Protons Triggers a Specific Cellular DNA Damage Response. Int. J. Radiat. Oncol. Biol. Phys..

[13] Quail D.F., Joyce J.A. (2013). Microenvironmental regulation of tumor progression and metastasis. Nat. Med..

[14] Wu T., Dai Y. (2017). Tumor microenvironment and therapeutic response. Cancer Lett..

[15] Merrick A., Errington F., Milward K., O’Donnell D., Harrington K., Bateman A., Pandha H., Vile R., Morrison E., Selby P. (2005). Immunosuppressive effects of radiation on human dendritic cells: Reduced IL-12 production on activation and impairment of naïve T-cell priming. Br. J. Cancer.

[16] Shiao S.L., Coussens L.M. (2010). The Tumor-Immune Microenvironment and Response to Radiation Therapy. J. Mammary Gland. Biol. Neoplasia.

[17] Ardura J.A., Rackov G., Izquierdo E., Alonso V., Gortazar A.R., Escribese M.M. (2019). Targeting Macrophages: Friends or Foes in Disease?. Front. Pharmacol..

[18] Poltavets A.S., Vishnyakova P.A., Elchaninov A.V., Sukhikh G.T., Fatkhudinov T.K. (2020). Macrophage Modification Strategies for Efficient Cell Therapy. Cells.

[19] Vishnyakova P., Poltavets A., Karpulevich E., Maznina A., Vtorushina V., Mikhaleva L., Kananykhina E., Lokhonina A., Kovalchuk S., Makarov A. (2021). The response of two polar monocyte subsets to inflammation. Biomed. Pharmacother..

[20] Guerriero J.L., Sotayo A., Ponichtera H.E., Castrillon J.A., Pourzia A.L., Schad S., Johnson S.F., Carrasco R.D., Lazo S., Bronson R.T. (2017). Class IIa HDAC inhibition reduces breast tumours and metastases through anti-tumour macrophages. Nature.

[21] Tsai C.-S., Chen F.-H., Wang C.-C., Huang H.-L., Jung S.-M., Wu C.-J., Lee C.-C., McBride W.H., Chiang C.-S., Hong J.-H. (2007). Macrophages from Irradiated Tumors Express Higher Levels of iNOS, Arginase-I and COX-2, and Promote Tumor Growth. Int. J. Radiat. Oncol. Biol. Phys..

[22] Genard G., Lucas S., Michiels C. (2017). Reprogramming of Tumor-Associated Macrophages with Anticancer Therapies: Radiotherapy versus Chemo- and Immunotherapies. Front. Immunol..

[23] Shan Y.-X., Jin S.-Z., Liu X.-D., Liu Y., Liu S.-Z. (2007). Ionizing radiation stimulates secretion of pro-inflammatory cytokines: Dose–response relationship, mechanisms and implications. Radiat. Environ. Biophys..

[24] Lambert L.E., Paulnock D.M. (1987). Modulation of macrophage function by gamma-irradiation. Acquisition of the primed cell inter-mediate stage of the macrophage tumoricidal activation pathway. J. Immunol..

[25] Shiga K., Hara M., Nagasaki T., Sato T., Takahashi H., Takeyama H. (2015). Cancer-Associated Fibroblasts: Their Characteristics and Their Roles in Tumor Growth. Cancers.

[26] Koosha F., Pourbagheri-Sigaroodi A., Bakhshandeh M., Bashash D. (2021). Low-dose radiotherapy (LD-RT) for COVID-19-induced pneumopathy: A worth considering approach. Int. J. Radiat. Biol..

[27] Ahn G.-O., Brown J.M. (2008). Matrix Metalloproteinase-9 Is Required for Tumor Vasculogenesis but Not for Angiogenesis: Role of Bone Marrow-Derived Myelomonocytic Cells. Cancer Cell.

[28] Doedens A.L., Stockmann C., Rubinstein M.P., Liao D., Zhang N., DeNardo D.G., Coussens L.M., Karin M., Goldrath A.W., Johnson R.S. (2010). Macrophage Expression of Hypoxia-Inducible Factor-1α Suppresses T-Cell Function and Promotes Tumor Progression. Cancer Res..

[29] Orr M.T., Lanier L.L. (2010). Natural Killer Cell Education and Tolerance. Cell.

[30] Gasser S., Orsulic S., Brown E.J., Raulet D.H. (2005). The DNA damage pathway regulates innate immune system ligands of the NKG2D receptor. Nature.

[31] Kim J.-Y., Son Y., Park S.-W., Bae J.-H., Chung J.S., Kim H.H., Chung B.-S., Kim S.-H., Kang C.-D. (2006). Increase of NKG2D ligands and sensitivity to NK cell-mediated cytotoxicity of tumor cells by heat shock and ionizing radiation. Exp. Mol. Med..

[32] Wu J., Liu T., Rios Z., Mei Q., Lin X., Cao S. (2017). Heat Shock Proteins and Cancer. Trends Pharmacol. Sci..

[33] Albakova Z., Siam M.K.S., Sacitharan P.K., Ziganshin R.H., Ryazantsev D.Y., Sapozhnikov A.M. (2021). Extracellular heat shock proteins and cancer: New perspectives. Transl. Oncol..

[34] Fiori M.E., Di Franco S., Villanova L., Bianca P., Stassi G., De Maria R. (2019). Cancer-associated fibroblasts as abettors of tumor progression at the crossroads of EMT and therapy resistance. Mol. Cancer.

[35] Kalluri R. (2016). The biology and function of fibroblasts in cancer. Nat. Rev. Cancer.

[36] Hellevik T., Pettersen I., Berg V., Winberg J.O., Moe B.T., Bartnes K., Paulssen R.H., Busund L.-T., Bremnes R., Chalmers A. (2012). Cancer-associated fibroblasts from human NSCLC survive ablative doses of radiation but their invasive capacity is reduced. Radiat. Oncol..

[37] Tsai K.K., Chuang E.Y.-Y., Little J.B., Yuan Z.-M. (2005). Cellular Mechanisms for Low-Dose Ionizing Radiation–Induced Perturbation of the Breast Tissue Microenvironment. Cancer Res..

[38] Wang L., Fossati P., Paganetti H., Ma L., Gillison M., Myers J.N., Hug E., Frank S.J. (2021). The Biological Basis for Enhanced Effects of Proton Radiation Therapy Relative to Photon Radiation Therapy for Head and Neck Squamous Cell Carcinoma. Int. J. Part. Ther..

[39] Chan J.S.K., Sng M.K., Teo Z.Q., Chong H.C., Twang J.S., Tan N.S. (2018). Targeting nuclear receptors in cancer-associated fibroblasts as concurrent therapy to inhibit development of chemoresistant tumors. Oncogene.

[40] Leung C.S., Yeung T.-L., Yip K.-P., Wong K.-K., Ho S.Y., Mangala L.S., Sood A.K., Lopez-Berestein G., Sheng J., Wong S.T. (2017). Cancer-associated fibroblasts regulate endothelial adhesion protein LPP to promote ovarian cancer chemoresistance. J. Clin. Investig..

[41] Su S., Chen J., Yao H., Liu J., Yu S., Lao L., Wang M., Luo M., Xing Y., Chen F. (2018). CD10^+^GPR77^+^ Cancer-Associated Fibroblasts Promote Cancer Formation and Chemoresistance by Sustaining Cancer Stemness. Cell.

[42] Ishii G. (2017). Crosstalk Between Cancer Associated Fibroblasts and Cancer Cells in the Tumor Microenvironment After Radiotherapy. eBioMedicine.

[43] Tommelein J., De Vlieghere E., Verset L., Melsens E., Leenders J., Descamps B., Debucquoy A., Vanhove C., Pauwels P., Gespach C.P. (2018). Radiotherapy-Activated Cancer-Associated Fibroblasts Promote Tumor Progression through Paracrine IGF1R Activation. Cancer Res..

[44] Diegeler S., Hellweg C.E. (2017). Intercellular Communication of Tumor Cells and Immune Cells after Exposure to Different Ionizing Radiation Qualities. Front. Immunol..

[45] Flickinger I., Rütgen B.C., Gerner W., Calice I., Tichy A., Saalmüller A., Kleiter M. (2013). Radiation up-regulates the expression of VEGF in a canine oral melanoma cell line. J. Veter. Sci..

[46] Pasi F., Facoetti A., Nano R. (2010). IL-8 and IL-6 bystander signalling in human glioblastoma cells exposed to gamma radiation. Anticancer Res..

[47] Baker D.G., Krochak R.J. (1989). The Response of the Microvascular System to Radiation: A Review. Cancer Investig..

[48] Denekamp J. (1984). Vascular Endothelium as the Vulnerable Element in Tumours. Acta Radiol. Oncol..

[49] Heckmann M., Douwes K., Peter R., Degitz K. (1998). Vascular Activation of Adhesion Molecule mRNA and Cell Surface Expression by Ionizing Radiation. Exp. Cell Res..

[50] Paris F., Fuks Z., Kang A., Capodieci P., Juan G., Ehleiter D., Haimovitz-Friedman A., Cordon-Cardo C., Kolesnick R. (2001). Endothelial Apoptosis as the Primary Lesion Initiating Intestinal Radiation Damage in Mice. Science.

[51] Wang J., Boerma M., Fu Q., Hauer-Jensen M. (2007). Significance of endothelial dysfunction in the pathogenesis of early and delayed radiation enteropathy. World J. Gastroenterol..

[52] Gujral D.M., Chahal N., Senior R., Harrington K.J., Nutting C.M. (2014). Radiation-induced carotid artery atherosclerosis. Radiother. Oncol..

[53] Hoving S., Heeneman S., Gijbels M.J., Poele J.A.T., Russell N.S., Daemen M., Stewart F.A. (2008). Single-Dose and Fractionated Irradiation Promote Initiation and Progression of Atherosclerosis and Induce an Inflammatory Plaque Phenotype in ApoE−/− Mice. Int. J. Radiat. Oncol..

[54] Russell N.S., Hoving S., Heeneman S., Hage J.J., Woerdeman L.A., de Bree R., Lohuis P.J., Smeele L., Cleutjens J., Valenkamp A. (2009). Novel insights into pathological changes in muscular arteries of radiotherapy patients. Radiother. Oncol..

[55] Kioi M., Vogel H., Schultz G., Hoffman R.M., Harsh G.R., Brown J.M. (2010). Inhibition of vasculogenesis, but not angiogenesis, prevents the recurrence of glioblastoma after irradiation in mice. J. Clin. Investig..

[56] Lin W.W., Karin M. (2007). A cytokine-mediated link between innate immunity, inflammation, and cancer. J. Clin. Investig..

[57] Chin M.S., Freniere B.B., Bonney C.F., Lancerotto L., Saleeby J.H., Lo Y.-C., Orgill D.P., Fitzgerald T.J., Lalikos J.F. (2013). Skin Perfusion and Oxygenation Changes in Radiation Fibrosis. Plast. Reconstr. Surg..

[58] Li C., Lu L., Zhang J., Huang S., Xing Y., Zhao M., Zhou D., Li D., Meng A. (2015). Granulocyte colony-stimulating factor exacerbates hematopoietic stem cell injury after irradiation. Cell Biosci..

[59] Reits E.A., Hodge J.W., Herberts C.A., Groothuis T.A., Chakraborty M., Wansley E.K., Camphausen K., Luiten R.M., De Ru A.H., Neijssen J. (2006). Radiation modulates the peptide repertoire, enhances MHC class I expression, and induces successful antitumor immunotherapy. J. Exp. Med..

[60] Hernandez C., Huebener P., Schwabe R.F. (2016). Damage-associated molecular patterns in cancer: A double-edged sword. Oncogene.

[61] Golden E.B., Pellicciotta I., Demaria S., Barcellos-Hoff M.H., Formenti S.C. (2012). The convergence of radiation and immunogenic cell death signaling pathways. Front. Oncol..

[62] Ebner D.K., Tinganelli W., Helm A., Bisio A., Yamada S., Kamada T., Shimokawa T., Durante M. (2017). The Immunoregulatory Potential of Particle Radiation in Cancer Therapy. Front. Immunol..

[63] Fernandez-Gonzalo R., Baatout S., Moreels M. (2017). Impact of Particle Irradiation on the Immune System: From the Clinic to Mars. Front. Immunol..

[64] Gameiro S.R., Jammeh M.L., Wattenberg M.M., Tsang K.Y., Ferrone S., Hodge J.W. (2014). Radiation-induced immunogenic modulation of tumor enhances antigen processing and calreticulin exposure, resulting in enhanced T-cell killing. Oncotarget.

[65] Liu Y., Yan W., Tohme S., Chen M., Fu Y., Tian D., Lotze M., Tang D., Tsung A. (2015). Hypoxia induced HMGB1 and mitochondrial DNA interactions mediate tumor growth in hepatocellular carcinoma through Toll-like receptor 9. J. Hepatol..

[66] Ran S. (2015). The Role of TLR4 in Chemotherapy-Driven Metastasis. Cancer Res..

[67] Luo Y., Chihara Y., Fujimoto K., Sasahira T., Kuwada M., Fujiwara R., Fujii K., Ohmori H., Kuniyasu H. (2013). High mobility group box 1 released from necrotic cells enhances regrowth and metastasis of cancer cells that have survived chemotherapy. Eur. J. Cancer.

[68] Apetoh L., Ghiringhelli F., Tesniere A., Obeid M., Ortiz C., Criollo A., Mignot G., Maiuri M.C., Ullrich E., Saulnier P. (2007). Toll-like receptor 4–dependent contribution of the immune system to anticancer chemotherapy and radiotherapy. Nat. Med..

[69] Xu J., Jiang Y., Wang J., Shi X., Liu Q., Liu Z., Li Y., Scott M.J., Xiao G., Li S. (2014). Macrophage endocytosis of high-mobility group box 1 triggers pyroptosis. Cell Death Differ..

[70] Pawaria S., Binder R.J. (2011). CD91-dependent programming of T-helper cell responses following heat shock protein immunization. Nat. Commun..

[71] Zeng G., Aldridge M.E., Tian X., Seiler D., Zhang X., Jin Y., Rao J., Li W., Chen D., Langford M.P. (2006). Dendritic Cell Surface Calreticulin Is a Receptor for NY-ESO-1: Direct Interactions between Tumor-Associated Antigen and the Innate Immune System. J. Immunol..

[72] Semenova I.B. (2016). Role of purinergic receptors in immune response. J. Microbiol. Epidemiol. Immunobiol..

[73] Abdolvahab M.H., Darvishi B., Zarei M., Majidzadeh-A K., Farahmand L. (2020). Interferons: Role in cancer therapy. Immunotherapy.

[74] Serrano A., Apolloni S., Rossi S., Lattante S., Sabatelli M., Peric M., Andjus P., Michetti F., Carri M.T., Cozzolino M. (2019). The S100A4 transcriptional inhibitor niclosamide reduces pro-inflammatory and migratory phenotypes of microglia: Implications for amyotrophic lateral sclerosis. Cells.

[75] Gebhardt C., Riehl A., Durchdewald M., Németh J., Fürstenberger G., Müller-Decker K., Enk A., Arnold B., Bierhaus A., Nawroth P.P. (2008). RAGE signaling sustains inflammation and promotes tumor development. J. Exp. Med..

[76] Bettum I.J., Vasiliauskaite K., Nygaard V., Clancy T., Pettersen S.J., Tenstad E., Mælandsmo G.M., Prasmickaite L. (2014). Metastasis-associated protein S100A4 induces a network of inflammatory cytokines that activate stromal cells to acquire pro-tumorigenic properties. Cancer Lett..

[77] Ichikawa M., Williams R., Wang L., Vogl T., Srikrishna G. (2011). S100A8/A9 Activate Key Genes and Pathways in Colon Tumor Progression. Mol. Cancer Res..

[78] Fini M.A., Elias A., Johnson R.J., Wright R.M. (2012). Contribution of uric acid to cancer risk, recurrence, and mortality. Clin. Transl. Med..

[79] Baracco E.E., Stoll G., Van Endert P., Zitvogel L., Vacchelli E., Kroemer G. (2019). Contribution of annexin A1 to anticancer immunosurveillance. OncoImmunology.

[80] Vacchelli E., Ma Y., Baracco E.E., Sistigu A., Enot D.P., Pietrocola F., Yang H., Adjemian S., Chaba K., Semeraro M. (2015). Chemotherapy-induced antitumor immunity requires formyl peptide receptor 1. Science.

[81] Widel M., Przybyszewski W.M., Cieslar-Pobuda A., Saenko Y.V., Rzeszowska-Wolny J. (2012). Bystander normal human fibroblasts reduce damage response in radiation targeted cancer cells through intercellular ROS level modulation. Mutat. Res. Mol. Mech. Mutagen..

[82] Desai S., Kobayashi A., Konishi T., Oikawa M., Pandey B.N. (2014). Damaging and protective bystander cross-talk between human lung cancer and normal cells after proton microbeam irradiation. Mutat. Res. Mol. Mech. Mutagen..

[83] Theelen W.S.M.E., Peulen H.M.U., Lalezari F., Van Der Noort V., De Vries J.F., Aerts J.G.J.V., Dumoulin D.W., Bahce I., Niemeijer A.-L.N., De Langen A.J. (2019). Effect of Pembrolizumab After Stereotactic Body Radiotherapy vs Pembrolizumab Alone on Tumor Response in Patients with Advanced Non–Small Cell Lung Cancer. JAMA Oncol..

[84] Grimaldi A., Simeone E., Giannarelli D., Muto P., Falivene S., Borzillo V., Giugliano F.M., Sandomenico F., Petrillo A., Curvietto M. (2014). Abscopal effects of radiotherapy on advanced melanoma patients who progressed after ipilimumab immunotherapy. Oncoimmunology.

[85] Chen M.-L., Wang F.-H., Lee P.-K., Lin C.-M. (2001). Interleukin-10-induced T cell unresponsiveness can be reversed by dendritic cell stimulation. Immunol. Lett..

[86] Allen C.P., Tinganelli W., Sharma N., Nie J., Sicard C., Natale F., King M.I., Keysar S.B., Jimeno A., Furusawa Y. (2015). DNA Damage Response Proteins and Oxygen Modulate Prostaglandin E2 Growth Factor Release in Response to Low and High LET Ionizing Radiation. Front. Oncol..

[87] Li M.O., Flavell R.A. (2008). TGF-β: A Master of All T Cell Trades. Cell.

[88] Vitti E.T., Parsons J.L. (2019). The Radiobiological Effects of Proton Beam Therapy: Impact on DNA Damage and Repair. Cancers.

[89] Bernier J., Hall E.J., Giaccia A. (2004). Radiation oncology: A century of achievements. Nat. Rev. Cancer.

[90] Mitteer R.A., Wang Y., Shah J., Gordon S., Fager M., Butter P.-P., Kim H.J., Guardiola C., Carabe-Fernandez A., Fan Y. (2015). Proton beam radiation induces DNA damage and cell apoptosis in glioma stem cells through reactive oxygen species. Sci. Rep..

[91] Crittenden M., Kohrt H., Levy R., Jones J., Camphausen K., Dicker A., Demaria S., Formenti S. (2015). Current Clinical Trials Testing Combinations of Immunotherapy and Radiation. Semin. Radiat. Oncol..

[92] Jr H.J.L., Zeng J., Rengan R. (2018). Proton beam therapy and immunotherapy: An emerging partnership for immune activation in non-small cell lung cancer. Transl. Lung Cancer Res..

[93] Cao M.D., Chen Z.D., Xing Y. (2004). Gamma irradiation of human dendritic cells influences proliferation and cytokine profile of T cells in autologous mixed lymphocyte reaction. Cell Biol. Int..

[94] Meng Y., Mauceri H.J., Khodarev N.N., Darga T.E., Pitroda S.P., Beckett M.A., Kufe D.W., Weichselbaum R.R. (2010). Ad.Egr-TNF and Local Ionizing Radiation Suppress Metastases by Interferon-β-Dependent Activation of Antigen-specific CD8+ T Cells. Mol. Ther..

[95] Lugade A.A., Moran J.P., Gerber S.A., Rose R.C., Frelinger J.G., Lord E.M. (2005). Local Radiation Therapy of B16 Melanoma Tumors Increases the Generation of Tumor Antigen-Specific Effector Cells That Traffic to the Tumor. J. Immunol..

[96] Lupu-Plesu M., Claren A., Martial S., N’Diaye P.-D., Lebrigand K., Pons N., Ambrosetti D., Peyrottes I., Feuillade J., Hérault J. (2017). Effects of proton versus photon irradiation on (lymph)angiogenic, inflammatory, proliferative and anti-tumor immune responses in head and neck squamous cell carcinoma. Oncogenesis.

[97] Huang D., Ding Y., Zhou M., Rini B.I., Petillo D., Qian C.-N., Kahnoski R., Futreal P.A., Furge K.A., Teh B.T. (2010). Interleukin-8 Mediates Resistance to Antiangiogenic Agent Sunitinib in Renal Cell Carcinoma. Cancer Res..

[98] Fujita Y., Okamoto M., Goda H., Tano T., Nakashiro K.-I., Sugita A., Fujita T., Koido S., Homma S., Kawakami Y. (2014). Prognostic Significance of Interleukin-8 and CD163-Positive Cell-Infiltration in Tumor Tissues in Patients with Oral Squamous Cell Carcinoma. PLoS ONE.

[99] Wu C.-T., Chen M.-F., Chen W.-C., Hsieh C.-C. (2013). The role of IL-6 in the radiation response of prostate cancer. Radiat. Oncol..

[100] Gordon K., Smyk D., Gulidov I. (2021). Proton Therapy in Head and Neck Cancer Treatment: State of the Problem and Development Prospects (Review). Sovrem. Tehnol. Med..

[101] Blanchard P., Gunn G.B., Lin A., Foote R.L., Lee N.Y., Frank S.J. (2018). Proton Therapy for Head and Neck Cancers. Semin. Radiat. Oncol..

[102] Galluzzi L., Vitale I., Warren S., Adjemian S., Agostinis P., Martinez A.B., Chan T.A., Coukos G., Demaria S., Deutsch E. (2020). Consensus guidelines for the definition, detection and interpretation of immunogenic cell death. J. Immunother. Cancer.

[103] Friedman E.J. (2002). Immune Modulation by Ionizing Radiation and its Implications for Cancer Immunotherapy. Curr. Pharm. Des..

[104] Diamond M.S., Kinder M., Matsushita H., Mashayekhi M., Dunn G.P., Archambault J.M., Lee H., Arthur C.D., White J.M., Kalinke U. (2011). Type I interferon is selectively required by dendritic cells for immune rejection of tumors. J. Exp. Med..

[105] Mirjolet C., Nicol A., Limagne E., Mura C., Richard C., Morgand V., Rousseau M., Boidot R., Ghiringhelli F., Noel G. (2021). Impact of proton therapy on antitumor immune response. Sci. Rep..

[106] Zhang F., Manna S., Pop L.M., Chen Z.J., Fu Y.-X., Hannan R. (2020). Type I Interferon Response in Radiation-Induced Anti-Tumor Immunity. Semin. Radiat. Oncol..

[107] Ando K., Fujita H., Hosoi A., Nakawatari M., Nakamura E., Kakimi K., Nakano T., Imai T., Shimokawa T. (2013). Effective Suppression of Pulmonary Metastasis in Combined Carbon Ion Radiation Therapy with Dendritic-Cell Immunotherapy in Murine Tumor Models. Int. J. Radiat. Oncol. Biol. Phys..

[108] Shimokawa T., Ma L., Ando K., Sato K., Imai T. (2016). The Future of Combining Carbon-Ion Radiotherapy with Immunotherapy: Evidence and Progress in Mouse Models. Int. J. Part. Ther..

[109] Gameiro S.R., Malamas A.S., Bernstein M.B., Tsang K.Y., Vassantachart A., Sahoo N., Tailor R., Pidikiti R., Guha C.P., Hahn S.M. (2016). Tumor Cells Surviving Exposure to Proton or Photon Radiation Share a Common Immunogenic Modulation Signature, Rendering Them More Sensitive to T Cell–Mediated Killing. Int. J. Radiat. Oncol..

[110] Kamada T., Tsujii H., Blakely E.A., Debus J., De Neve W., Durante M., Jäkel O., Mayer R., Orecchia R., Pötter R. (2015). Carbon ion radiotherapy in Japan: An assessment of 20 years of clinical experience. Lancet Oncol..

[111] Seshacharyulu P., Baine M.J., Souchek J., Menning M., Kaur S., Yan Y., Ouellette M.M., Jain M., Lin C., Batra S.K. (2017). Biological determinants of radioresistance and their remediation in pancreatic cancer. Biochim. Biophys. Acta.

[112] Gordon K., Gulidov I., Fatkhudinov T., Koryakin S., Kaprin A. (2022). Fast and Furious: Fast Neutron Therapy in Cancer Treatment. Int. J. Part. Ther..

[113] Wennerberg E., Vanpouille-Box C., Bornstein S., Yamazaki T., Demaria S., Galluzzi L. (2017). Immune recognition of irradiated cancer cells. Immunol. Rev..

[114] Zhang Z., Liu X., Chen D., Yu J. (2022). Radiotherapy combined with immunotherapy: The dawn of cancer treatment. Signal Transduct. Target. Ther..

[115] Uemura S., Demizu Y., Hasegawa D., Fujikawa T., Inoue S., Nishimura A., Tojyo R., Nakamura S., Kozaki A., Saito A. (2022). The comparison of acute toxicities associated with craniospinal irradiation between photon beam therapy and proton beam therapy in children with brain tumors. Cancer Med..

[116] Lautenschlaeger S., Iancu G., Flatten V., Baumann K., Thiemer M., Dumke C., Zink K., Hauswald H., Vordermark D., Mauz-Körholz C. (2019). Advantage of proton-radiotherapy for pediatric patients and adolescents with Hodgkin’s disease. Radiat. Oncol..

[117] Doyen J., Falk A.T., Floquet V., Hérault J., Hannoun-Lévi J.-M. (2016). Proton beams in cancer treatments: Clinical outcomes and dosimetric comparisons with photon therapy. Cancer Treat. Rev..

[118] Yuan T.-Z., Zhan Z.-J., Qian C.-N. (2019). New frontiers in proton therapy: Applications in cancers. Cancer Commun..

